# Oral health issues of young adults with severe intellectual and developmental disabilities and caregiver burdens: a qualitative study

**DOI:** 10.1186/s12903-021-01896-3

**Published:** 2021-10-18

**Authors:** Jihyun Lee, Juhea Chang

**Affiliations:** 1 Department of Dental Education and Dental Research Institute, School of Dentistry, Seoul National University, 101 Daehak-no, Jongno-gu, Seoul, 03080 South Korea; 2 National Dental Care Center for Persons with Special Needs, Seoul National University Dental Hospital, 101 Daehak-no, Jongno-gu, Seoul, 03080 South Korea

**Keywords:** Burdens, Caregiver, Intellectual disabilities, Oral health, Qualitative

## Abstract

**Background:**

Oral health maintenance is difficult to be achieved alone by patients with special needs and insufficient self-care skills. This study aims to investigate how the oral health issues of young adults with severe intellectual and developmental disabilities (IDD) affect caregiver burdens.

**Methods:**

A qualitative research method was employed with semi-structured interviews conducted with 14 maternal caregivers of patients with severe IDD. Eleven young adults had neurofunctional disorders and three had autism spectrum disorders. All recorded data were transcribed verbatim and subjected to thematic analysis.

**Results:**

Three themes emerged from the main agenda: predisposing oral dysfunction, home care challenges, and professional treatment barriers. The severity of the disabilities had an impact on oral disease risks that increased as patients aged. Participants indicated that, among the daily living activities of their patients, toothbrushing was a particular hardship due to their dysphagia and behavioral issues. Factors impacting on dental treatment indicated by caregivers included social, emotional, and financial circumstances.

**Conclusions:**

Dysphagia and behavioral issues of adult patients with severe IDD contributed to caregiver burdens in the dental care of the patients. Caregiver burdens and barriers to treatment were mutual factors hindering adequate interventions in dealing with dental problems of the patients.

**Supplementary Information:**

The online version contains supplementary material available at 10.1186/s12903-021-01896-3.

## Background

Persons with severe to profound intellectual and developmental disorders (IDD) need supervision in social settings and help with self-care activities [1]. These individuals have limited ability to communicate and often also have physical limitations. Oral health maintenance is a particularly important issue for this vulnerable population that experience both poor oral health and a high level of unmet treatment needs [2]. As in other health care areas, their oral health care is largely dependent on the knowledge, attitude, and practices of their caregivers. Moreover, their deficiency of self-advocacy; often being unable to report dental problems, reduces the chances of timely interventions. When the self-reporting of a patient is problematically obtained or regarded as unreliable, it is common to rely on the reports from other informants, most often parental caregivers, who are principally observant and knowledgeable about their children’s conditions [3]. Their accurate proxy-reports can have major positive impact on managing the oral health issues of the patients. Until recently, a vast majority of studies of oral health related proxy-reports have been focused on parental caregivers of young children [4–6], but rarely on those of adult children, although it is their dental problems that become complicated over time. Due to deficient communicative skills of the patients, caregivers are limited in their awareness and understanding of patients’ conditions, resulting in the existing and ongoing diseases becoming more aggravated. A previous study demonstrated that edentulousness of adults with intellectual disabilities (ID) was prevalent compared to their nondisabled counterparts (31% vs. 3% in 25–44 yrs) and those with more severe degrees of IDs showed even higher incidence [7].

Ascertaining oral health status, based on proxy-reports, has been largely conducted using self-administrated questionnaires. In a recent study by Chang [8], caregiver ratings and dentist assessments on the status of patients’ oral health were compared to indicate non-communicated problems of the adult patients with severe IDD. In the study, primary caregivers, who were family members with a high awareness of dental care, were presented with questionnaires; however, several questions were unanswered and subsequently left out of the outcome analysis. Consequently, only a limited number of variables were validated for relevant factors in these clinical circumstances. In such a case, a qualitative investigation approach can capture a wide range of information, allowing for an increased understanding of the targeted population [9, 10]. It is also a useful measure to generate questionnaire items by involving the population of interest to better reflect their perspectives and conditions [11]. Qualitative methods are also a beneficial tool in investigating dental experiences and circumstances within minority groups, bringing knowledge of the unknowns to the forefront [12]. In light of this, patients with severe IDD and their family caregivers as proxy-reporters present as a target population for the purpose of qualitative research. Through specifying the experiences of patients as well as caregivers, underlying causes and origins of patients’ oral health problems can be clarified to dental professionals. The aims of this study are: to explore the oral health issues of young adults with severe IDD experienced through their maternal caregivers and also identify the key factors that intensify the burdens on the caregivers in the maintenance of patients’ oral health.

## Methods

Qualitative research methodology was employed, specifically, that of thematic analysis, in order to render the detailed in-depth interview data into a set of core themes; “How oral health issues of young adult with severe IDD intensify the caregiver burdens in maintaining the oral health of the patients?” Thematic analysis is a popular, yet flexible method, of analyzing qualitative data that can be applied, which focuses on identifying patterned meaning across a dataset through recursive processes of generating, reviewing, defining and redefining themes [13]. We decided on and applied this method due to its flexibility and hybrid approach to inductive and deductive theme development, and to individual and social experiences.

### Study participants

A purposeful sampling was used to meet the research criteria. The purposeful sampling in a qualitative study is a sampling method employed for recruiting participants who can provide in-depth, rich and detailed understanding and insights into the phenomena under study. Two focus groups were formed, each comprising four women aged between 45 and 60, each a mother of a student of between 18 and 22 years old, currently attending a high school for special needs education suffering a severe degree of brain disorders. Further interviews were conducted with eight mothers, 47–59 years old, of children, age range 18–27 years who sought dental treatment under general anesthesia, due to a lack of cooperative skills, at Seoul National University Dental Hospital. The children with IDD were the patients who were receiving long term care from the author (JC). After the study goals were introduced and the interview outlines explained, the mothers agreed to participate in the interviews. For the focus groups, one of the interviewees contacted the mothers of children attending the above-mentioned school, since her child had graduated from the same school, and she had kept a strong connection with the school and the mothers. Demographic characteristics of the mothers and children are shown in Table 1. This study was approved by the institutional board of research of Seoul National University Dental Hospital (IRB No. CRI19001), and the written consent, based on GDPR guidelines, was obtained from each participant.

**Table 1:** Demographic characteristics of study participants

Participant ID	Mothers		Children						
	Age	Stay-at-home	Age	Sex	Disability type		Underlying causes	Need for assistance in daily living activities (1 = bathing, 2 = dressing, 3 = transferring, 4 = toileting, 5 = eating	Siblings
FG 1-1	45–60	yes	21	M	Brain disorder	Birth defect (craniosynostosis)		1,2,3,4,5	2 Sisters
FG 1-2		yes	19	F	Brain disorder	Genetic disorder (Angelman syndrome)		1,2,3,4,5	1 Brother
FG 1-3		yes	18	M	Brain disorder	Brain injury (onset:6 yrs)		1,2,3,4,5	1 Sister
FG 1-4		yes	22	F	Brain disorder	Urea cycle disorder		1,2,3,4,5	1 Sister
FG 2-1		yes	19	M	Brain disorder	Premature birth		1,2,3,4,5	2 Sisters
FG 2-2		yes	19	M	Brain disorder	Birth asphyxia		1,2,3,4,5	2 Sisters
FG 2-3		yes	19	F	Brain disorder	Birth asphyxia		1,2,3,4,5	1 Brother
FG 2-4		yes	20	F	Brain disorder	Birth asphyxia		1,2,3,4,5	None
Int 1	48	no	20	F	Brain disorder	Genetic disorder (Angelman syndrome)		1,2,3,4,5	1 Sister, 1 brother
Int 2	50	yes	27	M	Brain disorder	Cerebral palsy		1,2,3,4,5	1 Sister
Int 3	52	yes	27	M	Developmental disability	Autism		1	1 Brother
Int 4	48	yes	21	M	Developmental disability	Autism		1	1 Sister
Int 5	59	no	25	M	Developmental disability	Autism		1	1 Brother
Int 6	47	yes	18	F	Brain disorder	Birth asphyxia		1,2,3,4,5	None

*FG* focus group, *Int* interview

### Data collection

A topic guide was constructed to cover the outline of the study by a clinical dentist with qualitative research training (JC). The topic guide was reviewed by an experienced qualitative researcher (JL) and rehearsed prior to the meetings with participants. The interview questions were presented in Additional file 1. Ten interviews were conducted in total: six individual interviews with each participant and two group interviews with four participants in each. The time and places of interviews were chosen by the participants to avoid the interruption of caregiving activities. The interviews were performed in quiet and isolated meeting places at the hospital and the school. For the six individual interviews, one of the authors (JC) conducted the semi-structured interviews with open-ended questions. For the group interview, an interviewer with qualitative research training, who was neither an author nor a dentist, was engaged in the meeting to lead the discussion ensuring neutrality and non-bias. Interviews were digitally audio-recorded and lasted 21–75 min. Data were continually collected until no additional theme emerged, i.e., until theoretical saturation was reached.

### Data analysis

All the recoded interview data were transcribed verbatim, and then analyzed by reading each transcript thoroughly and repeatedly. The coding of transcripts and the development of categories were performed by one author (JC) and reviewed by the other author (JL) for analysts’ triangulation. Most of coding was agreed between the two researchers through ongoing discussions. The refined codes constitute sub-themes and themes with clarified definitions and examples of participants remarks. For the participants’ triangulation, the conduct of observation and member checking was undertaken. The authors’ understanding and interpretations were checked with participants to ensure their consistency with the subjects’ experiences and intentions, thus verifying the data and its interpretation. Finally, as the themes and findings were compiled, summarized, and any interplays among them were revealed, a conceptual model developed that depicts how oral health issues of patients with severe IDD affect the caregiver burdens. The consolidated criteria for reporting qualitative research (COREQ) were checked to ensure the comprehensiveness and credibility of this study [12].

## Results

### Themes

**Fig. 1:**
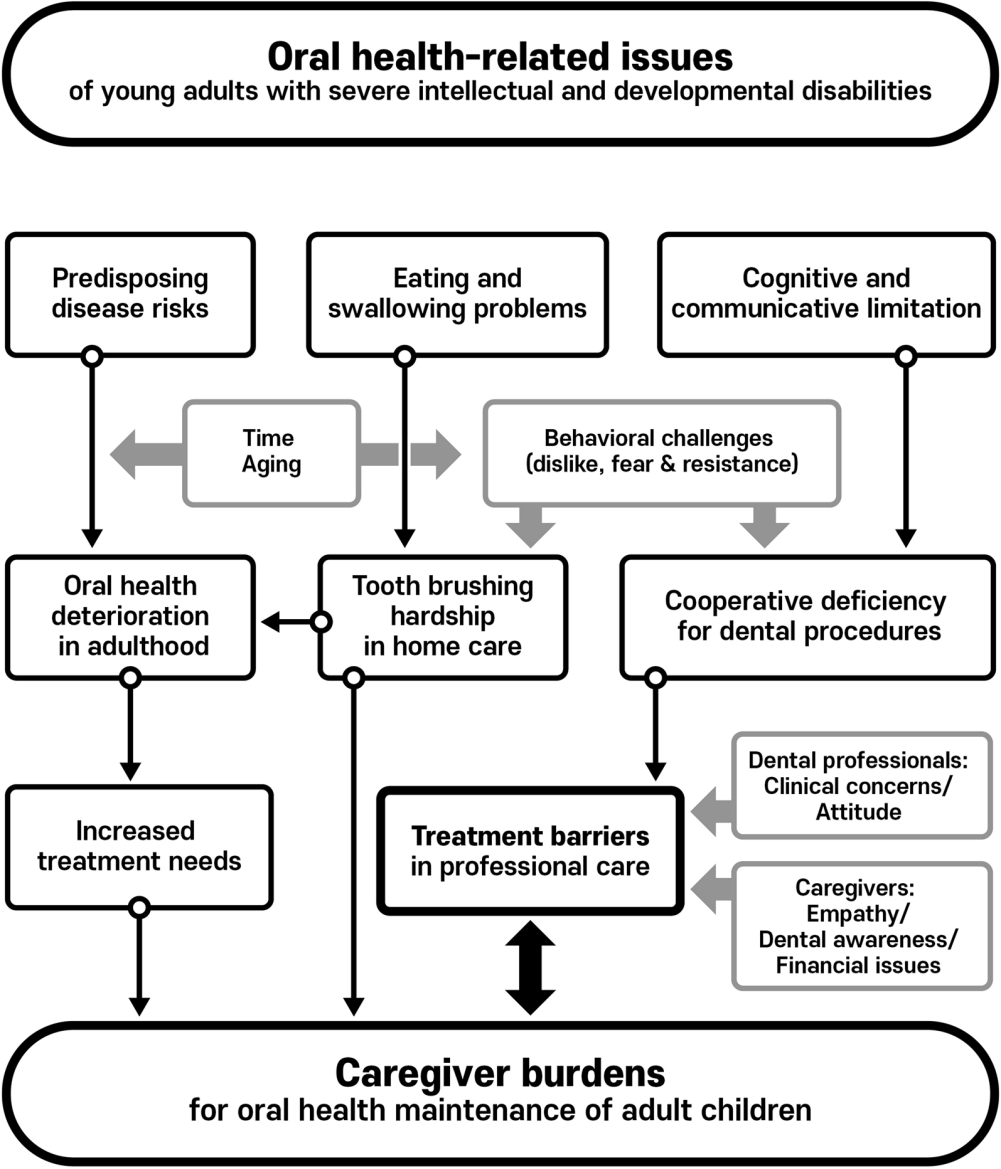
Findings of how oral health issues of patients with severe IDD affect the caregiver burdens

Three main themes were identified as key factors intensifying caregivers’ burdens in maintaining oral health of their children with IDD: (1) predisposing risk factors; (2) home care challenges; and (3) professional treatment barriers. Each theme was substantiated by individual sub-themes that are discussed in details below. Exemplary quotations under categorization are enlisted in Table 2. The framework depicted how the research findings were drawn in the study (Fig. 1).

**Table 2:** Themes and data categorization

Themes	Subthemes	Definition	Exemplary quotations
Predisposing oral dysfunction	Eating problems	Difficulty in chewing and swallowing	“He could swallow it, but only shallowly and then only once or twice, there was still a lot of food in his mouth. In addition, there remained the problem with choking.” (FG 1-3) “Even with soft foods like bananas, he doesn’t chew, rather he sucks it in his mouth before swallowing. Chewing is virtually impossible.” (FG 2-2)
	Abnormal diet and increased caries risk	Food impaction	“What I’m most concerned about is that despite cutting food into a small size for her, the food becomes stuck between her teeth. We can floss or pick the teeth, but, she cannot tell us what’s bothering her. The food will be there for days; and eventually she will be ended up having cavities.” (FG 2-3)
		Sugar uptake	“These kids are mostly on a liquid diet and I’m afraid that it is high in sugar.” (FG 2-4)
	Deteriorated deciduous dentition	Congenital defects	“When her teeth first came out, they were all black and rotten. I’ve heard that it might be something to do with the medicine she was taking at that time.” (FG 2-3)
		Baby bottle tooth decay	“She started having regular meals when she turned four. Before then, she was biting the milk bottle all the time.” (Int 1)
Home care challenges	Physical adversities	Rinsing and spitting	“When brushing teeth, it is natural that we hold the water in our mouth, but, for her, it’s not easy. She just swallows all of the toothpaste foam.” (FG 1-2) “When I finish his brushing, the toothpaste foam is completely gone; he ate it all.” (FG 1-3)
	Behavioral rejection	Dislike of untasteful objects	“The bristles go into the mouth and touch it where she doesn’t want to be touched. She has never got used to the feeling, even with brushes having thin, fine bristles.” (FG 1-4) “There may be differences as to the degree, but the truth is they all hate the tooth brush.” (FG 1-1) “Once I used a toothpaste which was not what I usually did, and he threw up all he had eaten… Kids on a liquid diet have a weak stomach.” (FG 2-2)
	Deficit of self-care	Caregiver responsibility	“Tooth brushing is much harder than washing his body. Giving him a bath is way easier.” (FG 1-3) “We let him brush by himself first, then to ensure that all spots are reached. We go over it again. He can’t do it properly—just a few quick swipes…” (Int 5) “I imagine it would be difficult for volunteer workers or school faculty to master it (toothbrushing), because our family finds it hard as well… I usually do it because his father feels pressured with me watching next to him.” (FG 1-1)
Professional treatment barriers	Cognitive impairment	Communicative limitation	“Because of the medication, he is insensitive to any types of pain and, of course when it does hurt, he doesn’t cry about it, just bear it. The fact that he can’t express what he’s feeling…, well, as a parent, I’m extremely sensitive about it.” (FG 2-2)
		Lack of understanding	“At the dental office, he saw many sharp looking instruments, and suddenly he was afraid that he’s going to get pinched and freaked out. We had to return home without doing anything.” (Int 5)
	Fear and resistance	Noise, smell, needles, mouth opening, physical restraint	“The sound of scratching is the most terrifying. Even me, I’m scared of it.” (Int 4) “The thought of opening his mouth and being tied down by force. Not to mention the frightening sounds.” (FG 2-1) “They hate opening their mouths. When they walk in the office, the first thing they see is people lying down, all with mouths gaping wide.” (FG 2-3) “An enormous fear of needles…, when he had to get shots, ah… I could write a book about it.” (Int 5)
	Rejection by and unwillingness of professionals	Attitude and rejection	“A volunteer doctor was there, he was somewhat disrespectful, played fast and loose, I didn’t want to go there again… We are extremely conscious about how our children are treated.” (FG 1-1) “(at the clinic) she behaved in an uncontrollable way, and the doctor refused to…” (FG 1-3)
		Concerns about clinical conditions	“Most local clinics cannot possibly manage these children.” (Int 2) “The staff were extremely anxious and recommended that we seek treatment at some other place.” (FG 2-4)
	Financial burdens	Cost	“Costs are the main issue. Most mothers are concerned and asking me about it” (Int 1)
			“(In dental care) the most desperate thing is… money.” (Int #4)
		Insurance coverage	“What I wish for most, … is financial support, and better insurance coverage. Then, the kids can come seek treatment as soon as they show the first sign of cavities” (Int 6)
	Emotional aspects of caregivers	Awareness	“Many children don’t visit the dental clinic at all. Their mothers take a glimpse at their teeth, and believe the teeth are fine, then the children don’t see the dentist.” (FG 2-4)
		Empathy	“There are kids who intensely, aggressively show signs of their dislike… the mothers are uncomfortable in such situations and feeling apprehensive about it…, I have never had such a heavy heart as when going to the dentist with him” (Int 3)

#### Predisposing oral dysfunction

##### Eating problems

All participants reported that their children were exhibiting comprehensive problems related to eating, drinking and swallowing. The children with severe brain disorders relied on a modified diet, such as mashed or softened foods dependent on individual circumstances. The children with autistic disorders showed behavioral patterns such as cramming and hastened eating and swallowing without proper chewing. There were considerable concerns raised in relation to dysphagia symptoms, since aspiration of food or fluid into the airways can increase the chances of both respiratory infection and asphyxiation.

“When she is eating something that she likes, she gulps it down with excitement… her swallowing is interrupted with frequent coughing. She can’t freely exercise tongue coordination. Her tongue is always busy pushing out food when she is really trying to take it in. And I’m continuously trying to push the food into her mouth with a spoon. It takes almost an hour for her to finish the meal.” (Int 6).

##### Abnormal diet and increased caries risk

Chewing and swallowing difficulties led the children to keep food remnants in their mouth without swallowing. Some children were fed a liquid diet, and the hidden sugar contained in their meals were concerns for the caregivers.

##### Dental caries/anomalies of the deciduous dentition

Some participants reported encountering congenital anomalies and caries development of deciduous teeth in their children. Children with severe brain disorders had experienced eating problems since birth and lived on prolonged bottle feeding. A number of participants acknowledged that they were overwhelmed by dealing with the adverse health conditions of their children and so paid relatively less attention to their teeth. Some attributed the experience of extended caries from an early age to be a turning point in making them aware of the importance of the oral health care of their children.

#### Home care challenges

##### Physiological adversities

A number of participants emphasized that teeth brushing was one of the most challenging tasks among the daily living activities of their children. The main challenge experienced during brushing was that children were neither able to hold water in their mouth nor to spit it out. Some caregivers hesitated to pressure their children into brushing their teeth, because they swallowed the toothpaste without rinsing and spitting out.

##### Behavioral rejection

Participants indicated that generally the children exhibited a strong dislike to teeth brushing and toothpaste.

“Once his mouth is opened, he consistently bites down to whatever’s inside the mouth. He cannot open his mouth enough, so, pushing a toothbrush into his mouth is pretty difficult, the toothbrush head is too big to reach deep inside the mouth, and moving the brush around to reach all parts of the mouth is a frustrating deal. We are telling him, “You’ve been doing this for 21 years, and you still can’t do it!” (FG 1-3).

##### Deficit of self-care

The participants in this study were the primary caregivers of their own children and felt more responsibilities for the daily oral hygiene maintenance among their other activities. Teeth brushing was a physically demanding job both for mothers and children, particularly, as mothers were becoming older, and children were growing up. Other family members did help the children in brushing their teeth but were not as competent in doing so as their mothers. Nevertheless, the mothers believed that it was essential to keep their children’s’ teeth clean and was a task that should not be ignored. If not properly done, the consequences would be serious, resulting in the need for dental treatment, a situation that worried the caregivers.

“When my kid is free from cavities, I believe that my helping with his teeth brushing worked out. It’s quite a big thing. I think nobody cannot trust these kids to take care of themselves alone…. Once they have cavities, the treatment is going to be a huge work. He can’t do that. I don’t want to even imagine about getting him to do…” (Int 5).

#### Professional treatment barriers

##### Cognitive impairment

Some participants were frustrated by that their children were unable to express their pain or discomfort by verbal communication. When the children exhibited unusually restless signs, the caregivers were not certain that dental problems were one of the causes. This more likely occurred when the caregivers were not aware of the children’s poor oral health conditions and worried about their potential dental problems. Even when they noticed the abnormal signs in the mouths of their children, they were reluctant to take their children to the clinic right at that moment. Their concerns were that the children would neither properly understand the reasons and nor be able to tolerate the procedures.

##### Fear and resistance

All participants indicated that their children had excessive fear of the environment of dental clinics. The children were particularly sensitive to the noise, smell, and visual images of sharp instruments, including needles. The children also refused to enter the office having caught sight of other patients lying with their mouths open. They perceived that they had to do the same thing once they were in.

##### Rejection and unwillingness of professionals

A few participants had experiences of being rejected by dental clinics when they sought treatment for their children. They were further frustrated when dental and medical professionals refused their treatment because of behavioral issues, predisposing diseases, current medication, breathing and swallowing dysfunctions and other underlying health conditions. The attitude of the professionals affected the caregivers’ feelings and discouraged them from seeking the needed treatments.

##### Financial burdens

The participants suggested that dental cost is another major concern of caregivers, particularly when their children have complicated dental problems brought on by delayed treatment. The children, already relying on various medical treatment caused the caregivers to be burdened with accumulated medical bills, and the addition of dental costs was regarded as more monetary burden on the health care of their children.

##### Caregiver motivation

A number of children had serious systemic health issues, making it difficult to prioritize their dental problems. Caregivers tended to neglect the dental problems of the children, particularly, when decayed teeth were not easily discernible. Emotional aspects of caregivers also contributed to a reluctance in seeking treatment for their children. When the children were strongly resistant to receiving the treatment, their caregivers prioritized the children’s feelings and were unwilling to bring hardship to their children in the form of dental treatment.

“We’ve been to almost all departments of the hospital, all except for the dentist, because I never wanted to go. It was too painful. When he was five or six, we went to the dental clinic, a nurse and I held on to him hard to keep him lying down, and I remember his sweat dripping down, it almost wet the doctor’s gown. Seeing my kid suffering so much drove me crazy. So, we delayed dental treatment for a couple of years…, I know, it is always better to try to prevent rather than be sorry later. But we were so afraid, truly afraid…” (Int 3).

## Discussion

This study applied a qualitative approach to elucidate the factors intensifying the caregivers’ burdens in the oral health care of the young adults with severe IDD. We selected primary maternal caregivers who were knowledgeable about the children’s circumstances and mainly assisted in their daily living activities. The participants indicated that toothbrushing was a particularly difficult task for their children, but they could not ignore it due to the fear of dental problems being potentiated. Treatment barriers were another serious stressor felt by caregivers that were further affected by social, emotional, and financial impacts.

All participants reported that their children experienced dysphagia-related issues. Eating and swallowing problems are prevalent among people with IDD, and the incidence and intensity of the problems increase with the severity of IDD [14]. Most children in this study had suffered a severe degree of neurofunctional disorders since birth and their daily living was entirely dependent on the caregivers’ assistance. As noted by our participants, toothbrushing was also affected by the patients’ oral motor disorders, experienced by most patients with IDD and regarded as a particular challenge among other daily cleaning activities. Children’s negative attitudes towards toothbrushing were barely alleviated as they grew up, while caregivers became increasingly exhausted by the adversity of the task. Participants also witnessed many other caregivers being helpless in this situation and gave-up toothbrushing, despite noticing their patients’ dental conditions becoming aggravated as a result. This study revealed how difficult was daily toothbrushing for patients with severe IDD, even by maternal caregivers who were highly motivated and resourceful in doing the task. Moreover, the patients’ dysphagia problems provided a physiological barrier to conventional dental treatments because of the patients’ difficulty in holing water in their mouth and withstanding the entire procedure while having to keep their mouth wide-opened. Many participants reported that their children had negative impressions through previous dental treatments, and sometimes were traumatized by the memories. The caregivers felt pressured to help their children with brushing and regarded it as a preventive measure against future treatment needs. In this regard, family caregivers may differ to professional ones in motivation and responsibility to maintain the oral hygiene of their patients.

Aging factors, it would seem, intensify the burdens of caregivers. Many participants reported that as their children with IDD entered adulthood, their behavioral patterns became more accentuated and less manageable. Moreover, risks of potential oral diseases tend to increase, due to consistent retention of food in the mouth, incompetent brushing and flossing, and a dry mouth resulting from long-term medications and physiological dysfunctions [15]. Many caregivers noted that when children with IDD stick to unhealthy dietary patterns, such as dependence on liquid or soft food, frequent snacking, and preference for a high-sugar diet, it becomes more difficult to modify their habits as they grow up, because they resist more than they did in childhood. A systematic literature review identified that maternal burden was higher when the adult with IDD had poorer physical health, and that disability-related expenses increased with time [14]. Dental cares impose an additional financial load that are difficult to prioritize over main health care issues. Unresolved dental problems at a young age of children with IDD will exacerbate over time, leading to heightened treatment needs such as emergency department visits and hospitalization under general anesthesia [16].

Behavioral issues caused from IDD were found to be one of the main factors hindering the undertaking of professional dental care. Insufficient communicative skills and reduced cognitive abilities of the patients presented challenges in three distinct ways. Firstly, patients experience restricted awareness and exhibit intolerance towards dental procedures and instead, exhibit fear and rejection in unwanted situations. Secondly, caregivers have limited knowledge of children’s symptoms and are uncertain as to whether they should arrange dental visits based on their perception. In addition, caregivers were often discouraged in seeking treatments through negative experience from previous visits or concerns about being rejected and refused treatment. Even when the patients are permitted treatment, they are frustrated with the children’s resisting and suffering during the procedures. Third and finally, dental professionals are faced with difficulties in preoperative screenings, determining problem severity, and performing other evaluations and often give up initiating actual treatments [17].

The qualitative investigation of this study demonstrated how challenging was the task of maintaining oral health of patients with IDD in daily lives for caregivers. Considering the large heterogeneity of population with IDD at different life stages, it is problematic to allocate representative groups into the investigation as in quantitative studies. In accordance with patient circumstances, caregivers are also subjected to a diversity of background variables. However, in order to compensate, a theoretical framework was constructed elucidating the cause and impact of oral-health issues from disabilities connected to the caregiver burdens (Fig. 1). One useful application of qualitative research is the development of items for questionnaires, with individual interviews and focus groups being the two predominant methods of collecting the perspectives from populations of interests [11]. Previously, caregiver burdens had been evaluated using self-registered questionnaires that were only limitedly associated with oral health maintenance of patients. In a study of Japanese nursing home caregivers, oral health-related caregiver burdens were verified using nine questions selected from general caregiver burdens (Burden Index of Caregivers, BIC-11) [18]. In the study, each four domains were constructed from only a single, or pair of items, and inclusion of more relevant variables would be required for construct validity. Other studies in the USA attempted to correlate caregiver burdens and preventive dental care of their children with IDD, and were mainly focused on socioeconomic circumstances [15, 16]. It was shown that the severity of burdens was in accordance with the severity of disabilities and social disadvantages. However, other barriers against preventive dental cares remained even for patients with adequate insurance coverage.

This study supports the view that caregiver burdens and treatment barriers are mutual issues hampering the appropriate intervention on the dental problems of this vulnerable population. One possible limitation of the study, requiring cautious interpretation, is its partial generalizability of the finding to some similar context. The goal of qualitative studies is not to generalize but rather to provide a rich, contextualized understanding of some aspect of the issues under study, through the intensive study of particular cases, which implies a need for quantitative studies with representative samples. A mixed approach to the issue would examine the generalizability of the findings to broader populations, even though the severities and characteristics of IDD are extremely diverse. A further limitation of this study is its cross-sectional design, suggesting a need for well-designed prospective investigations as IDD individuals and their caregivers become older. Nevertheless, it is our hope that this study contributes in clarifying attributing factors that signify the burdens and barriers caregivers are faced with so that solutions to relieve their difficulties can be further explored with the aim of meeting the dental treatment needs of patients with severe IDD.

## Conclusions

Dysphagia problems and behavioral issues of adult patients with severe IDD were shown to intensify caregiver burdens in administering dental care for the patients. Caregiver burdens and treatment barriers were correlated issues hindering adequate interventions in dealing with dental problems of the patients.

## Supplementary Information

**Additional file 1.** Questionnaires used in the study.

## Data Availability

The datasets used and/or analyzed during the current study are available from the corresponding author on reasonable request.
