# Supplementary material for: Oral health issues of young adults with severe intellectual and developmental disabilities and caregiver burdens: a qualitative study
Source: BMC Oral Health. 2021 Oct 18;21:538. doi: 10.1186/s12903-021-01896-3 (PMC8524865; doi:10.1186/s12903-021-01896-3)
Supplement: Supplementary file 1 — Additional file 1. Questionnaires used in the study. [file 12903_2021_1896_MOESM1_ESM.docx]

Supplemental table: Interview questions used in the study

| **Interview Questions** | | | |
| --- | --- | --- | --- |
| **Category** | **Subcategory** | **Check items** | **Example questions** |
| **Demographics** | Patients | Age | How old is your child? |
|  |  | Sex | Is your child your daughter or son? |
|  |  | Disabilities | What kind of disabilities does your child have? |
|  |  | Systemic health | Does she/he have any systemic health problems?  Does she/he take any medication? |
|  |  | Lifestyle | Are there any places she/he regularly attends?  How does she/he manage her/his own daily living activities?  Are there any specific difficulties on her/his daily living activities? |
|  | Caregivers | Age | How old are you? |
|  |  | Caregiving type | Who else is taking care of the child?  Does the child have any siblings? |
|  |  | Occupation | Are you a stay-at-home mother or employed? |
| **Clinical factors** | Caries risk factors | Diet (snack) | Does your child have any favorite foods/snacks? How often does she/he have her/his meals/snacks? |
|  |  | Dental visit | Does your child visit the dentist regularly?  Has your child ever been given fluoride application at a dental clinic? |
|  |  | Caries experience | Does your child have any cavities, or did she/he ever have cavities? |
|  |  | Salivary flow | Do you think that your child has enough saliva in her/his mouth? |
|  |  | Oral hygiene | How does your child brush her/his teeth? |
|  | Oral health related factors | Missing teeth | Does your child have any missing teeth? |
|  |  | Periodontal condition | Does your child show any sign of bleeding on her/his gum when brushing her/his teeth? |
|  |  | Malocclusion | Do you find the teeth of your child well-aligned? |
| **Others** | Barriers to dental care | Dental visit  Obstacles | If you have/had difficulties in bringing your child to a dental clinic, what are/were your concerns? |
|  |  | Treatment needs | What treatment do you think that your child needs now? |
